# Alfalfa Polysaccharide Alleviates Colitis by Regulating Intestinal Microbiota and the Intestinal Barrier Against the TLR4/MyD88/NF-κB Pathway

**DOI:** 10.3390/nu17183001

**Published:** 2025-09-19

**Authors:** Shaokai La, Muhammad Abaidullah, Hao Li, Yalei Cui, Boshuai Liu, Yinghua Shi

**Affiliations:** 1 College of Animal Science and Technology, Henan Agricultural University, Zhengzhou 450046, China; lskmymailbox@163.com (S.L.); drabaidullah@henau.edu.cn (M.A.); lhmymailbox@163.com (H.L.); yaleicui423@henau.edu.cn (Y.C.); 2 Henan Key Laboratory of Innovation and Utilization of Grassland Resources, Zhengzhou 450046, China; 3 Henan Forage Engineering Technology Research Center, Zhengzhou 450046, China

**Keywords:** alfalfa polysaccharide, anti-inflammatory, mucosal barrier, *Parabacteroides distasonis*, colitis

## Abstract

**Background/Objectives**: Ulcerative colitis (UC) pathogenesis involves gut barrier dysfunction, dysregulated immune responses, and gut microbiota imbalance. Alfalfa polysaccharide (APS), a bioactive compound with immunomodulatory potential, remains underexplored in intestinal inflammation. While APS exhibits anti-inflammatory properties in vitro, its in vivo efficacy, mechanisms, and ability to restore gut microbiota and barrier integrity in UC are unclear. This study aims to investigate the treatment effect of APS on dextran sulfate sodium (DSS)-induced colitis in mice and confirm its prebiotic potential. **Methods**: A mouse model of ulcerative colitis was induced by DSS. RNA sequencing, Western blotting, the terminal deoxynucleotidyl transferase dUTP nick end labeling technique, and an immuno-histochemical technique were used to study the mechanism of action by which APS at different dosages relieves DSS-induced colitis. **Results**: The findings show that APS alleviated the symptoms of colitis in mice given DSS, improved the gut morphology, heightened goblet cells production, increased the levels of IL-10 and IL-22, decreased the levels of TNF-α, IL-1β, and IL-6, and prevented the activation of the TLR4/MyD88/NF-κB pathways. Additionally, they maintained the integrity of the intestine by enhancing the expression of the mucins MUC2 and MUC5AC and by increasing the amounts of ZO-1, Occludin, and Claudin-1 proteins. Moreover, APS supported the growth of probiotic bacteria, including *unclassified_f_lachnospiraceae*, *Parabacteroides*, *Alistipes,* and *Mucispirillum*, and in particular, *Parabacteroides distasonis*, which is strongly associated with decreased pro-inflammatory cytokine through the inhibition of the TLR4-MyD88-NFκB pathways. **Conclusions**: APS can be used as a new type of prebiotic to improve UC by regulating intestinal flora and enhancing intestinal barrier function against the TLR4-MyD88-NFκB pathway.

## 1. Introduction

In recent years, the incidence of ulcerative colitis (UC) has become increasingly severe on a global scale, with its morbidity showing a continuous upward trend. UC is a rather complex, chronic, and unspecified intestinal inflammatory disorder, and its pathogenesis involves multiple pathogenic factors, such as the disorder of the intestinal mucosal immune system, the imbalance of the functions of immune cells, and genetic susceptibility [1,2]. The gut microbiota is highly vital for the human body, playing an extremely crucial role in various aspects such as digestion, nutrient absorption, immune regulation, resistance against pathogens, metabolic regulation, and influencing mental health [3]. Relevant studies in recent years have found that the composition and characteristics of the gut microbiota in UC patients are strikingly different from those of normal individuals. Specifically, the amount of harmful bacteria in the intestine, like *Clostridioides difficile*, *Shigella,* and *Escherichia coli* [4], relatively increases, while the amount of certain characteristic beneficial bacteria, like *Akkermansia*, *Bifidobacteriaceae* [5], and *Roseburia* [6], relatively decreases. Meanwhile, it induces chronic inflammation and metabolic dysfunction and ultimately affects the intestinal immune system. Therefore, research based on the gut microbiota has emerged as a prominent focus within the realm of UC research. The pursuit of strategies to steer the transformation of the gut microbiota in a direction beneficial to the human body represents a crucial domain that is currently undergoing intensive exploration.

In addition, as a chronic inflammatory disease, the onset and progression of UC are intricately linked to numerous inflammation-associated factors, with intestinal barrier impairment being a particularly significant one. It has been reported that the intestinal physical barrier serves as the primary and crucial line of defense against diverse potential pathogens. Being a one-of-a-kind selective barrier system peculiar to the intestine, it has extremely important functions. It can effectively prevent exogenous antigens from causing damage to the body and, thus, represents an irreplaceable and vital part of sustaining the homeostasis of the internal environment of the intestine [7]. However, studies have found that the intestinal barrier dysfunction induced by UC will not only damage the integrity of the intestinal mucosa, making deleterious agents within the intestine, like bacteria and toxins, more likely to penetrate the intestinal barrier and enter the blood circulation, thereby triggering a systemic inflammatory response, but also affect the normal absorption of nutrients by the intestine, resulting in malnutrition in patients, such as weight loss and anemia, and will further aggravate the symptoms of enteritis, forming a vicious cycle [8]. Therefore, suppressing inflammation and enhancing intestinal barrier function are of utmost significance as strategies for the clinical prevention and treatment of UC.

Currently, traditional immunosuppressive drugs are frequently utilized in the clinical management of UC. While these medications demonstrate significant short-term therapeutic outcomes, long-term use can lead to obvious systemic side effects that are prone to recurrence after discontinuation [9]. Consequently, the development of novel, highly efficient, and low-toxicity therapeutic drugs is of paramount importance for the prevention and treatment of UC. Reports indicate that over the past few decades, numerous plant-derived polysaccharides, including Tremella fuciformis polysaccharide [10], Astragalus polysaccharide [11], and Lycium barbarum polysaccharide [12], have been employed in the prevention and treatment of UC with remarkable effects. Known as the “king of forage grass”, alfalfa, a globally cultivated leguminous forage rich in bioactive compounds, has been traditionally used in herbal medicine for immune modulation [13]. Its primary active component, Alfalfa polysaccharide (APS), has been confirmed in in vitro experiments to alleviate oxidative damage and inhibit the generation of inflammatory cytokines. It achieves this by regulating the MAPK/p38 and NF-κB signaling pathway and increasing the cell viability of macrophages [14]. In addition, APS also has anti-tumor [15], lipid-lowering [16], antioxidant, and immunomodulatory [17] effects. Such effects of APS are exactly what is needed for the prevention of UC. In particular, given the significant roles of other plant polysaccharides in the prevention and treatment of UC, we speculate that APS could serve as a novel prebiotic to effectively prevent UC. Nevertheless, so far, no such report has emerged. In this experiment, a mouse UC model triggered by dextran sulfate sodium (DSS) was utilized to investigate the therapeutic impact of APS on UC and the underlying mechanism.

## 2. Materials and Methods

### 2.1. Materials and Reagents

The APS used in the study was supplied by Shanghai Winherb Medical Science Co., Ltd. (Shanghai, China) and had a purity of 95.7%. DSS, with a molecular weight ranging from 36 to 50 kilodaltons, was supplied from MP Biomedicals (Santa Ana, CA, USA). 5-Aminosalicylic Acid (5-ASA), with a purity exceeding 98%, was bought from ACMEC Biochemical Technology Co., Ltd. (Shanghai, China). Hematoxylin and eosin (H&E) staining, Alcian blue (AB) staining, 4% paraformaldehyde, and the primary antibodies, including MUC2 and MUC5AC, were procured from Wuhan Servicebio Technology Co., Ltd. (Wuhan, China). The enzyme-linked immunosorbent assay (ELISA) kits for mice IL-1β, IL-6, TNF-α, IL-10, IL-22, ZO-1, Occludin, and Claudin-1 were provided by Shanghai Enzyme Linked Biotechnology Co., Ltd. (Shanghai, China). TUNEL Apoptosis Detection Kit I, POD (MK1025) was purchased from Wuhan Boster Biological Technology Co., Ltd. (Wuhan, China). ChamQ Universal SYBR qPCR Master Mix and HiScript^®^ III RT SuperMix for qPCR (+gDNA wiper) were purchased from Nnanjing Vazyme Biotech Co., Ltd. (Nanjing, China). PierceTM Rapid Gold BCA Protein Assay Kit was procured from Thermo Fisher Scientific-CN (Cleveland, OH, USA). RIPA working fluid [containing Protease and phosphatase inhibitor, RIPA Lysis Buffer, and Phenylmethylsulfonyl fluoride (PMSF)] was obtained from Beyotime Biotechnology Co., Ltd. (Shanghai, China). NFκB-p65, MyD88, TLR4, and Glyceraldehyde-3-phosphate dehydrogenase (GAPDH) mouse monoclonal antibodies were supplied by Wuhan Sanying Biotechnology Co., Ltd. (Wuhan, China).

### 2.2. Experimental Design

**Figure 1:**
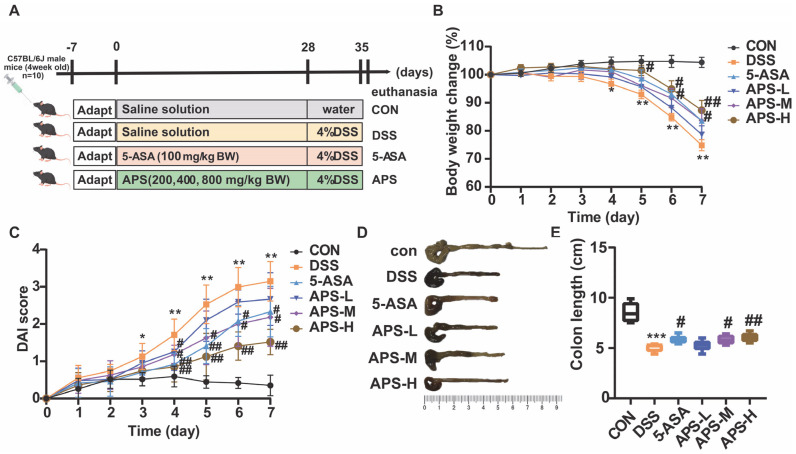
Experimental schematic diagram (**A**); APS alleviated the clinical symptoms of DSS-induced UC mice (**B**–**E**). (**B**) Body weight change. (**C**) Disease activity index (DAI) score. (**D**) Typical images of colon length for each category. (**E**) Colon length. All data are displayed as mean ± SEM (*n* = 10). * *p* < 0.05, ** *p* < 0.01, and *** *p* < 0.001 in comparison to the CON group; ^#^ *p* < 0.05 and ^##^ *p* < 0.01 in comparison to the DSS group.

Altogether, 60 male SPF-grade C57BL/6J mice aged four weeks were procured from Liaoning Changsheng Biotechnology Co., Ltd. (Liaoning, China). All the experimental mice were raised in the animal laboratory of Henan Agricultural University and were provided with sufficient food and water without any restrictions. At the same time, the standard laboratory environmental conditions, including humidity (50% ± 5%) and temperature (22 ± 2 °C), were also carefully maintained. Following a week of acclimatization and feeding to minimize stress-related physiological variations, all mice, which were individually housed in individually ventilated cages, were assigned at random to 6 separate groups equally (*n* = 10 per group). The number of mice in each group was comparable to a published study [18]. As demonstrated in Figure 1A, the mice groups included the control group (CON, normal diet), DSS treatment group (DSS, normal diet), 5-ASA treatment group (100 mg/kg of 5-ASA), APS-L treatment group (APS low-dose diet, 200 mg/kg of APS), APS-M treatment group (APS middle-dose diet, 400 mg/kg of APS), and APS-H treatment group (APS high-dose diet, 800 mg/kg of APS). Mice were given saline, APS [17], and 5-ASA [19] for 35 days. Among them, APS and 5-ASA were dissolved or suspended in normal saline for intragastric administration to ensure accurate and consistent delivery of dosages. Mice were given 4% DSS water to induce colitis, except the Con treatment group, from day 28 to 35, which is a well-established protocol for triggering acute intestinal inflammation. On the 36th day, mouse blood was collected into 1.5 mL vacuum tubes without anticoagulation by orbital blood sampling and centrifuged for 15 min at 4 °C 3000× *g* to extract serum, which was subsequently stored at −80 °C pending examination to preserve biomarker integrity. All mice were euthanized by placement in a sealed euthanasia chamber pre-filled with transparent CO_2_. Their colon lengths were recorded as a macroscopic indicator of inflammation, and colon tissue and stool samples were collected. A 4% paraformaldehyde solution was used to fix the distal colon tissue (about 1 cm) to maintain tissue morphology for follow-up histopathological and immuno-histochemical analysis. At the same time, another 1 mm^3^ colon tissue block was taken and placed in the EP tube of an electron microscope fixation solution for fixed preservation at 4 °C to enable ultrastructural examination. Fecal specimens and the remaining colon tissues were kept at −80 °C to prevent the degradation of molecular components for subsequent examination. All procedures, including dosing and sample collection, were conducted at a consistent daily time to minimize circadian-driven variability and ensure uniform treatment conditions, thereby reducing potential systematic bias.

### 2.3. Disease Activity Index (DAI) Evaluation

During the induction of colitis, the changes in the body weight of the mice were documented every day. Additionally, the rate of body weight loss, the presence of bloody stools, and stool morphology were observed and counted. These parameters were used to evaluate the DAI, which reflects the seriousness of colitis in the mice. Specifically, DAI = (weight loss rate + bloody stool + stool morphology)/3 [17]. To calculate the body weight loss, the percentage variation between the body weight at the start (day 0) and the body weight on every day after DSS treatment was utilized.

### 2.4. Histological Examination

The colonic tissues were immobilized in 4% paraformaldehyde solution for a minimum of 24 h. Afterwards, paraffin infiltration and embedding were performed after dehydration with different gradients of alcohol. Subsequently, 4 μm paraffin cuts of colon were prepared for H&E staining, followed by observation of colon tissue damage using an optical microscope for tissue pathology scoring, as previously described [18].

### 2.5. Alcian Blue Staining

According to the manufacturer’s instructions, 4 μm paraffin slices of colon tissue were stained with Alcian blue, and mucin-producing goblet cells were noticed and evaluated via an optical microscope.

### 2.6. Apoptosis Detection Test

Terminal deoxynucleotide transferase (TdT)-mediated d-UTP terminal labeling staining was employed to detect the apoptosis level in colon tissue. Briefly, the prepared colon tissue slices were treated with 3% H_2_O_2_ at room temperature for 25 min, then digested with Proteinase K at 37 °C for 5–10 min. The slices were labeled with TdT, DIG-d-UTP, and Labeling Buffer for 2 h and then washed with TBS, and sealing liquid was added to stand in an ambient environment for 30 min. The sections were subjected to biotinized anti-digoxin antibodies at 37 °C for 30 min before cleaning with TBS. SABC diluent was added to the slices at 37 °C for 30 min, and then they were cleaned with TBS again. After that, colon slices were subjected to staining using 3,3′-diaminobenzidine tetrahydrochloride (DAB), followed by slightly re-staining with hematoxylin, and a microscope observation of apoptosis.

### 2.7. Transmission Electron Microscopy (TEM) Analysis

The collected 1 mm^3^ colon specimens were immobilized in 2.5% glutaraldehyde (4 °C) for 24 h and subsequently rinsed 3 times utilizing 0.1M phosphate buffer PB with a pH of 7.4. The specimens were immobilized in 1% osmic acid at ambient temperature for 2 h. Subsequently, they were washed 3 times with 0.1M phosphate buffer PB of pH 7.4. The tissues were sequentially dehydrated through a graded ethanol series (30%, 50%, 70%, 80%, 95%, 100% twice)**,** followed by two changes of acetone, and embedded in SPI-Pon 812 epoxy resin (CAS 90529-77-4, SPI). The resin was obtained after the embedded plate was polymerized in an oven at 60 °C for 48 h. The resin was sliced with an ultra-thin micrograph (Leica UC7, Leica, Heerbrugg, Switzerland) and stained with a 2% uranium acetate saturated alcohol solution and 2.6% lead citrate solution to avoid light and carbon dioxide, respectively. Under a transmission electron microscope (HT7800/HT7700, Hitachi, Tokyo, Japan), the images were scrutinized and compiled for analysis.

### 2.8. Immuno-Histochemical Assay

As previously described, immuno-histochemical staining was used to examine mucin expression (MUC2 and MUC5AC) in colon tissue. Briefly, paraffin slices of the colon were dewaxed to water using xylene, followed by antigen repair using pH 8.0 EDTA buffer. After 3 PBS (pH 7.4) washes, the sections were incubated in 3% hydrogen peroxide liquid and placed in incubation at ambient temperature in the absence of light for 25 min to block endogenous peroxidase. Thereafter, 3% bovine serum albumin was added under ambient temperature and incubated for 30 min to achieve blocking. Next, the slices were treated by adding the primary antibody mixed with a certain proportion of PBS. The slices were then incubated flat in a wet box at 4 °C throughout the night. After the slices had been washed 3 times with PBS, the corresponding secondary antibody was added and remained under treatment for 50 min. Finally, the colonic tissue specimens were subjected to DAB staining, re-stained with hematoxylin, and observed under an optical microscope.

### 2.9. RNA Extraction and q-PCR

The mRNA levels of cytokines IL-1β, IL-6, TNF-α, IL10, TLR-4, MyD88, and NF-κB p65 in colon tissues were detected by Real-time PCR. Colon tissues underwent RNA extraction via the Trizol method. The NanoDrop 2000 nucleic acid protein analyzer (Thermo Fisher Scientific Inc., Wilmington, DE, USA) was used to evaluate RNA content and concentration. GAPDH can be utilized as an internal reference gene. The primers (primer sequences in Table S1) were developed and synthesized by Shangya Biotechnology Co., Ltd. (Zhengzhou, China). The extracted RNA samples were subjected to a reverse transcription process in accordance with the kit instructions. Next, qPCR was conducted using the LightCycler 96 real-time fluorescence quantitative PCR apparatus (Roche, Basel, Switzerland) in accordance with the directions provided by the manufacturer for the ChamQ Universal SYBR qPCR Master Mix. 2^−ΔΔCT^ was utilized to assess the mRNA expression level of the target gene.

### 2.10. Western Blot Assay

The colonic tissue was made homogeneous and then cleaved by a RIPA working fluid. After a 30 min ice bath, the lysate was centrifuged at 12,000 rpm for 5 min at 4 °C. Thereafter, the collected supernatant was the total protein solution. Colon tissue-derived proteins were separated and transferred onto PVDF membranes by means of 10% to 12% SDS-PAGE. The transformed PVDF membrane was placed in an appropriate amount of 5% skim milk powder for shaking and sealing for 1 h. Then, the primary antibody was added and incubated at 4 °C overnight. The membrane was then treated with the secondary antibody for 1 h. The freshly prepared ECL mixture was dripped onto the protein side of the membrane for color development to detect protein bands after TBST washing. The optical density value of the target strip was analyzed using an ImageJ software (v1.53q) processing system, and the result was normalized to GAPDH.

### 2.11. Biochemical Indices in Serum and Colon Analysis

The cytokines IL-1β, IL-6, TNF-α, IL-10, and IL-22 were measured in serum, while the tight junction proteins ZO-1, Occludin, and Claudin-1 were measured in colon tissue using ELISA kits, as recommended by the manufacturer.

### 2.12. DNA Extraction and Metagenome Sequencing

According to the requirements of metagenomic sequencing, 4 mice from each group were randomly selected for DNA extraction and metagenomic sequencing. As recommended by the manufacturer, the E.Z.N.A.^®^ soil DNA Kit (Omega Bio-tek, Norcross, GA, USA) was employed to obtain total bacterial DNA from colon tissue. The NanoDrop-2000 UV-vis spectrophotometer (Thermo Scientific Inc., Wilmington, DE, USA) was used to determine DNA concentrations, and 1% agarose gel electrophoresis was utilized to assess DNA quality. A metagenomic library was established with NEXTflexTM Rapid DNA-Seq (Bioo Scientific Inc., Austin, TX, USA). The Illumina NovaSeq 6000 (Illumina Inc., San Diego, CA, USA) sequencing platform was utilized for metagenomic sequencing at Shanghai Meiji Biomedical Technology Co., Ltd. (Shanghai, China).

### 2.13. Microbiome Analysis

Utilizing the original sequencing data, fastp (v0.20.0) was employed for data quality control, trimming off low-quality reads (length < 50 bp, average base quality < 20), as well as reads containing N bases, so as to obtain the high-quality sequences needed for subsequent analysis. BWA software (v 0.7.9a) was employed to map reads to host DNA sequences and remove highly/similar contaminated reads. The optimized sequences were assembled using MEGAHIT v1.1.2 [19], which relies on the succinct de Bruijn graphs principle. By selecting contigs with good assembly performance (≥300 bp), gene prediction was performed on the results using Prodigal v2.6.3 (https://github.com/hyattpd/Prodigal, accessed on 31 July 2022). Genes having nucleic acid lengths of at least 100 bp were chosen and transformed into amino acid sequences. A total of 9,032,803 genes were identified. Using CD-HIT v4.6.1 at 90% identity and coverage, predicted gene sequences from samples were clustered, generating a non-redundant set of 1,615,360 intact genes. High-quality reads from each sample were aligned to the gene set using the highly efficient SOAPaligner v2.21 with 95% identity. Gene abundance information was then calculated accordingly. Diamond v0.8.35 was utilized to perform two independent annotations: (1) taxonomic annotation by aligning against the Non-Redundant Protein Sequence Database (NR database) to obtain species classification based on the associated taxonomic information, following the convention prefixes denote taxonomic ranks: k__ (Kingdom), p__ (Phylum), c__ (Class), o__ (Order), f__ (Family), g__ (Genus), s__ (Species), and sp_ (unclassified species within a genus); (2) functional annotation by aligning against the Kyoto Encyclopedia of Genes and Genomes (KEGG) database (version 94.2). The parameters of BLASTP were configured to have an expected value of 1 × 10^−5^. This annotation process aimed to annotate bacterial gene sets and the KEGG gene profile. The amino acid sequences of the non-redundant gene collection were matched with the carbohydrate-active enzyme (CAZyme) database (v5.0) by employing hmmscan (https://www.ebi.ac.uk/Tools/hmmer/search/hmmscan, accessed on 31 July 2022) to annotate the profile of CAZyme.

To show the diversity and plenty of microbial communities, the Chao1, Shannon, and Simpson indices were determined with Mothur v1.30.2 [20]. The differences and similarities between groups of bacteria at the species level were illustrated through Principal Co-ordinates Analysis (PCoA). The Vegan package of R software (V3.6.0) was employed to examine the disparities in the microbial community structure between different treatments via ANOSIM. The relative abundances of phyla, genera, and species were respectively presented by barplots and heatmaps, which were created using R software (v3.3.1). The differences between the four groups were analyzed and identified by employing the Wilcoxon rank-sum test method. Linear discriminant analysis effect size (LEfSe) and linear discriminant analysis (LDA) were used to determine the species-level differential enrichment of colonic bacteria, microbial KEGG pathways, and microbial CAZymes between the groups. The association between the microbiota and tight junction proteins and colonic cytokines, respectively, was examined using Redundancy analysis (RDA).

### 2.14. Statistical Analysis

Before all the tests were conducted, the experimental personnel involved were unaware of the grouping of the mice. All the data from the experimental animals showed no abnormalities, and the data generated were all used for statistical analysis. Graph Pad Prism 7 and SPSS 21 were utilized for the data analysis. The data is shown as means ± Standard error of mean (SEM). To evaluate group differences, the one-way analysis of variance (ANOVA) was utilized. It is considered that *p*-values less than 0.05 (*p* < 0.05) are statistically significant.

## 3. Results

In this study, a mouse colitis model was induced using 4% DSS. The aim was to investigate the dose-dependent effect of APS in alleviating colitis in mice, and 5-ASA was used as the positive control drug.

### 3.1. APS Mitigated the Symptoms of Colitis

The underlying pathological symptoms in UC mice included colon shortening, weight loss, elevated DAI scores, and significant pathological alterations. In this study, the model mice experienced diarrhea, blood in the stool, weight loss, and a marked rise in DAI while receiving DSS therapy. However, in a dose-dependent way, APS alleviated the aforementioned symptoms (Figure 1B,C). One significant indicator of the severity of colitis brought on by DSS is a shorter colon. Compared to the control group, the DSS group’s colon length was shorter, as Figure 1D illustrates. Mice given DSS showed significantly longer colons after APS-H treatment (Figure 1E). Overall, these findings indicated that APS can effectively prevent mice from DSS-induced colitis.

### 3.2. APS Maintains Gut Integrity

**Figure 2:**
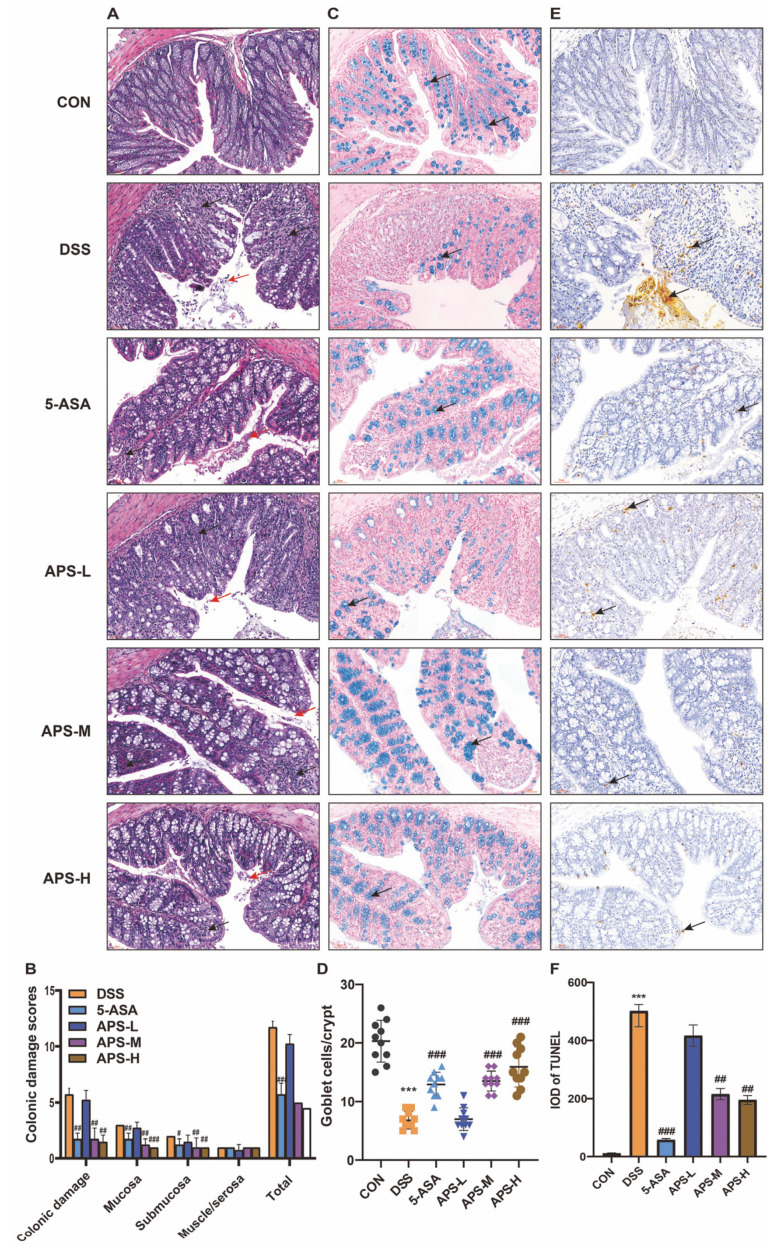
Prophylactic APS administration improved colonic integrity. (**A**) Typical hematoxylin and eosin (H&E)-stained colonic microscopic images (Scale bars: 50 μm) (*n* = 10). The red arrows denote epithelial shedding and the black arrows indicate inflammatory cell infiltration. (**B**) Colonic damage scores. (**C**) Typical images of the inner mucus layer of colonic sections stained with Alcian blue (AB) (Scale bars: 50 μm). The black arrows denote goblet cells. (**D**) Calculation of goblet cells in the colon (*n* = 10). (**E**) Representative TUNEL-stained pictures of colonic sections (Scale bars: 50 μm) (*n* = 3). The black arrow indicate apoptotic cells. (**F**) The integrated optical density (IOD) of TUNEL-positive expression in the colon tissues of each group. All data were displayed as mean ± SEM. *** *p* < 0.001 in comparison to the CON group; ^#^ *p* < 0.05, ^##^ *p* < 0.01, and ^###^ *p* < 0.001 in comparison to the DSS group.

It is commonly believed that UC is caused by damage to the intestinal mucosa. Its pathogenesis is linked to the weakening of gut epithelial barrier function and increased gut permeability [7]. UC was evaluated by means of previously defined macroscopic damage scoring [18]. Histological analyses demonstrated that the control group’s mucosal epithelium was intact and showed no signs of erosion. Moreover, there was no epithelial degeneration, and the glands of the lamina propria were neatly aligned. After being induced by DSS, severe colonic injuries appeared in the mice, including damage to the epithelial layer, infiltration of inflammatory cells in the mucosa and submucosa, destruction of the crypt structure, and loss of goblet cells. However, the supplementation of APS and positive drugs could significantly alleviate the colonic tissue damage and the loss of goblet cells in mice with colitis (Figure 2A–D). Furthermore, we found that the supplementation of APS effectively prevented the increase in the colon damage scores and the loss of goblet cells caused by DSS in a dose-dependent manner (Figure 2B,D), which further demonstrated that the epithelium of the colonic tissue was more complete and healthier after APS supplementation. Additionally, the TUNEL assay was used to examine epithelial apoptosis. In the mice with colitis induced by DSS, APS therapy significantly decreased the amount of apoptosis in the colonic epithelium, indicating that APS protected the epithelial barrier and prevented cell death (Figure 2E,F). By preserving intestinal integrity and inhibiting colonic tissue apoptosis in dose-dependent ways, the aforementioned findings demonstrated that APS was beneficial in the treatment of UC.

### 3.3. APS Restores the Tight Junctions and Enhances Protein Production

**Figure 3:**
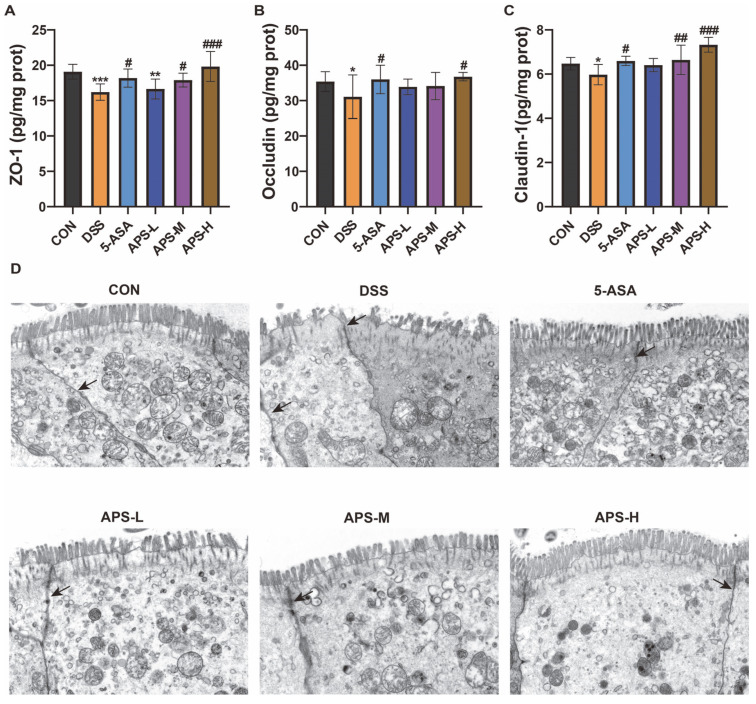
Prophylactic APS administration improved intestinal barrier function. Concentrations of ZO-1 (**A**), Occludin (**B**), and Claudin-1 (**C**) in the colon (*n* = 10). (**D**) Illustrations of the ultrastructure of colonocytes using transmission electron microscopy (TEM) (Scale bars: 2 μm) (*n* = 3). All data are displayed as mean ± SEM. * *p* < 0.05, ** *p* < 0.01, and *** *p* < 0.001 in comparison to the CON group; ^#^ *p* < 0.05, ^##^ *p* < 0.01, and ^###^ *p* < 0.001 in comparison to the DSS group.

Tight junction proteins maintain the intestinal mucosal barrier’s integrity [21]. According to the ELISA results, the ZO-1, Occludin, and Claudin-1 protein levels of the DSS group were significantly lower than those of the CON group. However, the protein levels of the 5-ASA and APS groups were restored to diverse magnitudes; the APS-H group, however, demonstrated an enhanced recovery performance and considerably raised the levels of ZO-1 (Figure 3A), Occludin (Figure 3B), and Claudin-1 (Figure 3C) proteins. The structural integrity of tight junctions was further examined using TEM. The DSS group presented with increased intercellular distance, loss of brush borders, and shortened apical–junctional complexes, when compared to the CON group (Figure 3D). In contrast, the 5-ASA and APS groups exhibited intensive and regular microvilli, and tight junctions were seen most apically. Based on the previously mentioned benefits of APS in treating UC caused by DSS, our results showed that APS-H had a significantly higher ameliorative effect on gut inflammation than any other group. As a result, APS-H was selected for further examination.

### 3.4. APS Restores MUC2 and MUC5AC Secretions

**Figure 4:**
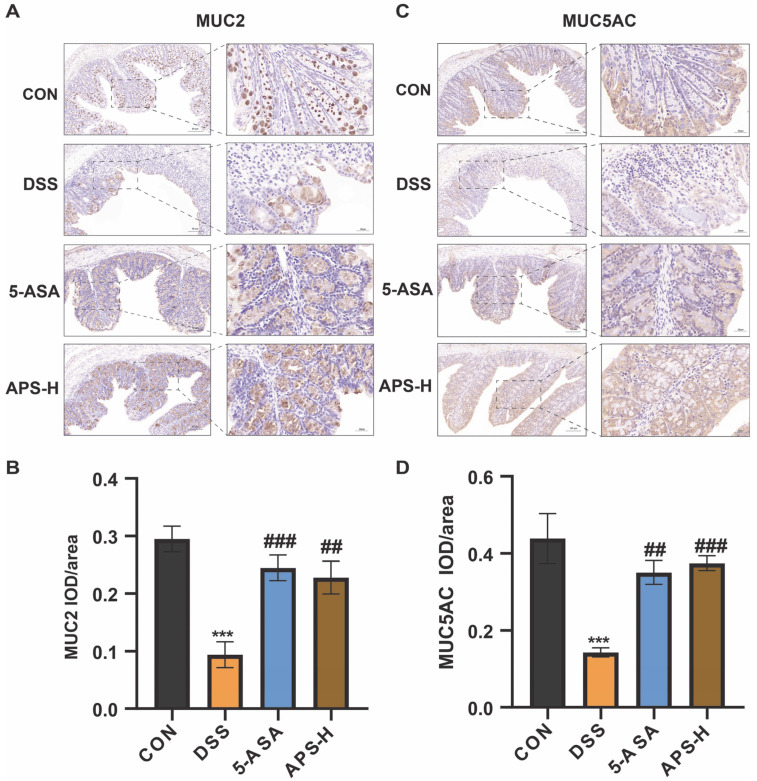
Prophylactic APS administration improved mucosal barrier function. (**A**) Immuno-histochemical analysis of MUC2 in colonic tissues (scale bar: 50 μm and 20 μm) (*n* = 3). (**B**) IOD/area was observed for MUC2 counts by immuno-histochemical staining. (**C**) Immuno-histochemical analysis of MUC5A C in colonic tissues (scale bar: 50 μm and 20 μm) (*n* = 3). (**D**) IOD/area was observed for MUC5AC counts by immuno-histochemical staining. *** *p* < 0.001 in comparison to the CON group; ^##^ *p* < 0.01, and ^###^ *p* < 0.001 in comparison to the DSS group.

Mucins like MUC2 and MUC5AC, which are essential for the operation of the mucus barrier, maintain the layer of mucus in the colon. The expression of MUC2 and MUC5AC was markedly down-regulated in the colon tissues of UC patients. This is associated with the severity and activity of UC [22]. Using immuno-histochemical labeling, we quantified the expression of the distal colon’s colonic mucus layer. When compared to the CON group, we discovered that DSS significantly reduced mucin expression (Figure 4). Interestingly, we found that MUC2 and MUC5AC expression was higher in the APS-H group than in the DSS group. The findings demonstrate that the down-regulation of MUC2 and MUC5AC caused by DSS was considerably reduced by APS-H. Drawing from the aforementioned data, we deduce that APS-H demonstrates potent mucus protective action by enhancing mucin secretion and thickening the mucus layer.

### 3.5. Protective Effect of APS-H Against Inflammation Through TLR4, MyD88, and the NF-κB Pathway

**Figure 5:**
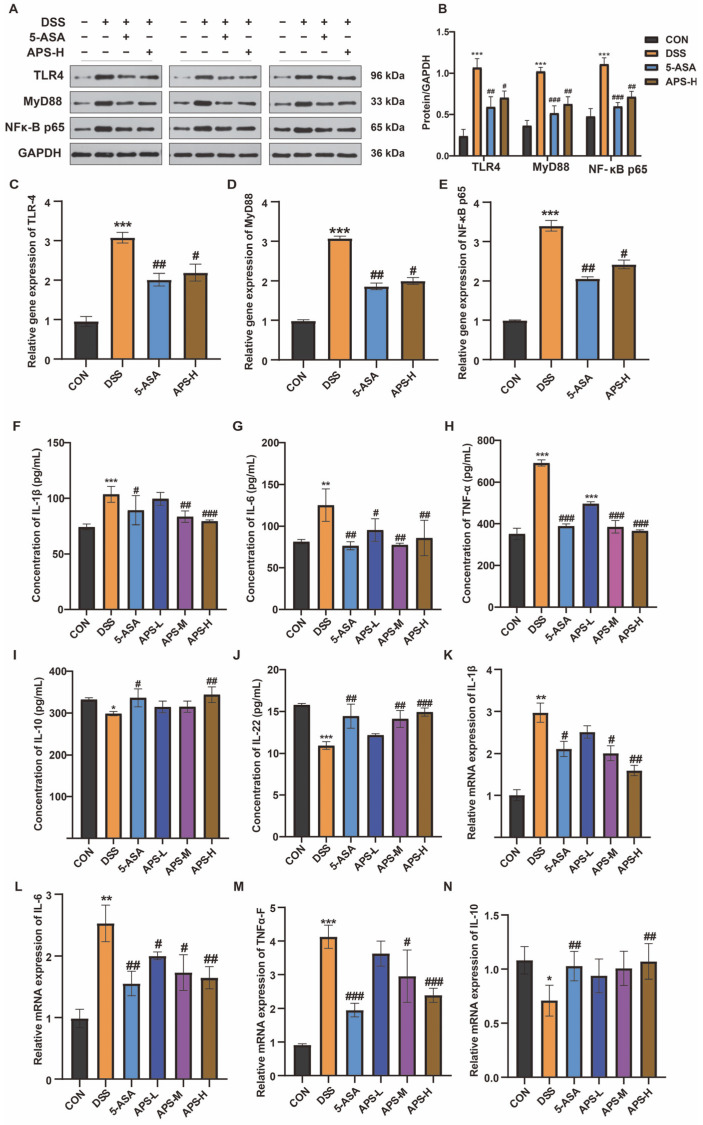
Prophylactic APS administration blocked the inflammatory pathways and ameliorated gut inflammation. (**A**) Protein levels of TLR4, MyD88, and NF-κB p65 in the colon were measured by Western blot (*n* = 3). (**B**) Quantification of band intensity using ImageJ (x64) software. (**C**–**E**) The relative gene expression levels of TLR-4, MyD88, and NF-κB P65 in the colon were measured by RT-qPCR (*n* = 6). Concentrations of IL-1β (**F**), IL-6 (**G**), TNF-α (**H**), IL-10 (**I**), and IL-22 (**J**) in the serum (*n* = 6). The relative mRNA expression levels of IL-1β (**K**), IL-6 (**L**), TNF-α (**M**), and IL-10 (**N**) in the colon (*n* = 6). All data are displayed as mean ± SEM. * *p* < 0.05, ** *p* < 0.01, and *** *p* < 0.001 in comparison to the CON group; ^#^ *p* < 0.05, ^##^ *p* < 0.01, and ^###^ *p* < 0.001 in comparison to the DSS group. GAPDH: Glyceraldehyde-3-phosphate dehydrogenase.

TLRs are thought of as bridges that change how the host immune system and gut bacteria interact. TLR4 levels have been found to be greater in UC patients [23]. Through the adaptor MyD88, TLR4 stimulation recruits its downstream, activating NF-κB and raising the levels of many inflammatory factors, such as TNF-α and IL-1β. NF-κB, MyD88, and TLR4 protein levels in the colon were measured with the aim of evaluating the impact of APS-H on gut inflammation brought on by DSS. DSS increased the activation of TLR4, MyD88, and the NF-κB pathway at the protein level, whereas APS-H administration led to a marked decrease in these protein levels (Figure 5A,B). Simultaneously, the relative mRNA expressions of NF-κB, MyD88, and TLR4 in the colon were noticeably increased in the DSS group compared with those in the remaining groups (Figure 5C–E). We observed the levels of three pro-inflammatory cytokines, namely IL-6, IL-1β, and TNF-α, in the serum and the mRNA expressions in the colon tissue proteins. The APS-H-treated colitis mice had down-regulated blood levels of IL-6, TNF-α, and IL-1β (Figure 5F–H), whereas the DSS-treated mice had increased serum levels of these cytokines. Meanwhile, APS-H supplementation further decreased the markedly increased gene expressions of these cytokines in the colon of the DSS-induced mice (Figure 5K–M). Additionally, APS-H strengthened the anti-inflammatory actions against mice having colitis induced by DSS (Figure 5I,J,N). The serum levels of IL-10 (Figure 5I) and IL-22 (Figure 5J), as well as the relative mRNA expressions of IL-10 (Figure 5N), were considerably lower in the DSS mice compared with those of APS-H mice. These findings demonstrate that APS-H could alleviate inflammation stresses in the gut by inhibiting the TLR4/MyD88/NF-κB pathway and its downstream cytokine production.

### 3.6. Alfalfa Polysaccharide Improves Intestinal Flora Imbalance

**Figure 6:**
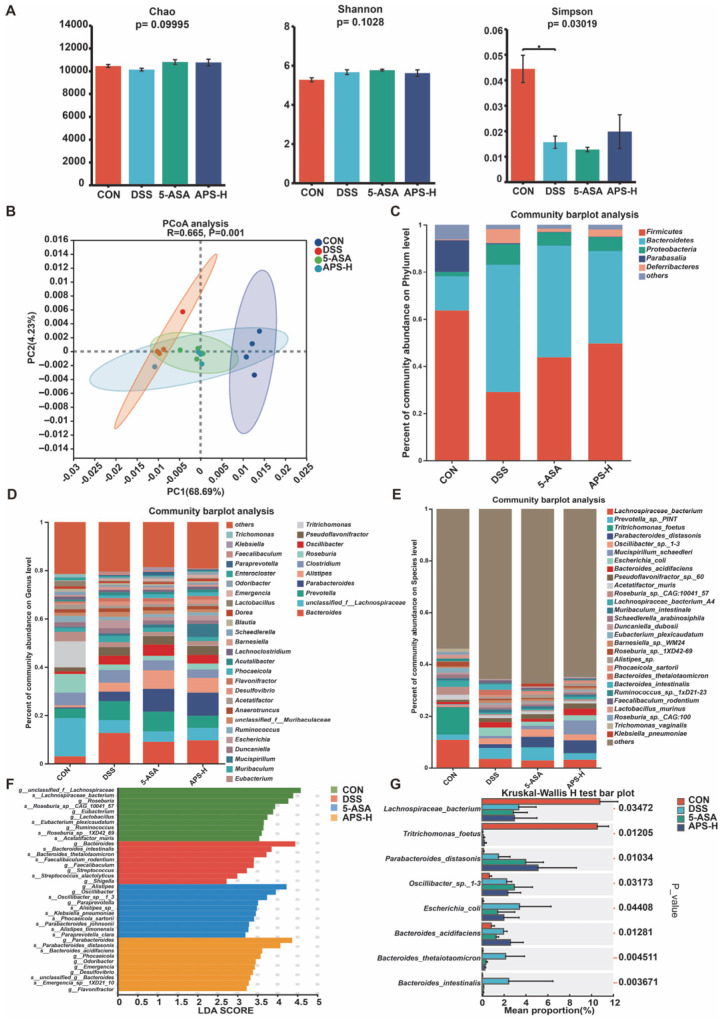
Prophylactic APS administration altered the composition of gut microbiota. Colon samples from the Con, DSS, 5-ASA, and APS-H groups were compared using metagenomics. (**A**) The α-diversity (Chao, Shannon, and Simpson index) of the colonic microbiota across four mouse groups. (**B**) Principal Co-ordinates Analysis (PCoA) plot showing the β-diversity of the gut microbiome. The gut microbial community is displayed at the phylum (**C**) and genus levels (**D**). (**E**) Community heatmap assessment of 30 species at the species level. (**F**) Linear discriminant analysis effect size (LEfSe) analysis of colonic bacteria differential enrichment at the species level (linear discriminant analysis [LDA] > 2.5). (**G**) Differential analysis among these four groups at the species level.

In order to investigate whether APS can change the composition of gut flora, we performed metagenomic sequencing of fecal samples from mice in the CON, DSS, 5-ASA, and APS-H groups. We mainly studied the disparities in the overall composition and specific differences of the intestinal microbial communities among the four groups, especially at the species level. Firstly, as illustrated in Figure 6A, there were no significant differences in the Chao and Shannon indices of α-diversity among the groups. However, the Simpson index values in the DSS group were lower than those in the CON group. The PCoA of colonic flora based on Euclidean distance was employed to assess the difference in overall flora structure among the CON, DSS, 5-ASA, and APS-H groups. According to Figure 6B, there were notable distinctions in the composition and profusion of flora among the groups, and the distance between the 5-ASA and APS-H groups and CON was smaller than that between the DSS group and the CON group. These data show that APS has changed the structure of gut flora.

To further explore the changes in microbial composition, we compared the relative abundance of dominant phyla, prevailing genera, and major species in each group, especially the change in taxa abundance caused by APS. In Figure 6C, Firmicutes, Bacteroidetes, Proteobacteria, Parabasalia, and Deferribacteres were the dominant phyla among the four groups at the phylum level. In contrast to the CON group, the comparative abundances of Bacteroidetes and Proteobacteria in the DSS, 5-ASA, and APS-H groups were elevated, whereas that of Firmicutes was reduced. In comparison to the DSS group, the APS group reduced the number of Bacteroidetes.

The analysis of the barplot diagram (Figure 6D) at the genus level showed that, in comparison with the CON group, the relative profusion of *Bacteroides*, *Prevotella*, *Clostridium, Oscillibacter*, *Pseudoflavonifractor Escherichia*, and *Desulfovibrio* was markedly augmented, while the relative profusion of *unclassified_f_lachnospiraceae*, *Roseburia*, *Tritrichomonas*, *Eubacterium,* and *Ruminococcus* was markedly reduced in the DSS group. Compared with the DSS group, APS-H supplementation has markedly augmented the relative profusion of *Parabacteroides*, *unclassified_f_lachnospiraceae*, *Alistipes*, and *Mucispirillum*.

Additionally, Figure 6E displayed the distribution of fecal bacteria in all samples at the species level. The clusterization analysis of the top 30 species in abundance revealed that although the three groups shared some overlapping locations, these species could separate each set of samples (Figure 6E). The heatmap analysis (Figure 6E) at the species level indicated that the dominant microorganism species in the DSS group were different from those in the CON group, APS-H group, and 5-ASA group. Most of the dominant species in the healthy tissue samples belong to Firmicutes, while most of the prevailing species in the DSS group belong to Proteobacteria. The dominant species in the 5-ASA and APS groups belong to the Parabacteroides. Furthermore, LDA and LEfSe were employed to examine the biomarkers among the four treatment groups (Figure 6F). The LDA findings showed that there were 10 distinguishing features in the CON group. Among them, *Lachnospiraceae_bacterium*, *Roseburia*_*sp*__*CAG*_*10041*_*57*, *Eubacterium_plexicaudatum*, *Roseburia*_*sp*__*1XD42_69,* and *Acetatifactor_muris* were the main species-level microorganisms. The species with high LDA scores in the DSS group were *Bacteroides_intestinalis*, *Bacteroides_thetaiotaomicron*, *Faecalibaculum_rodentium*, and *Streptococcus_alactolyticus*. There were six characteristic microorganisms in the 5-ASA group at the species level, which were *Oscillibacter*_*sp*_*_1*_*3*, *Klebsiella_pneumoniae*, *Phocaeicola_sartorii*, *Parabacteroides_johnsonii*, *Alistipes_timonensis*, and *Paraprevotella_clara*. However, the APS-H group only had three characteristic microorganisms: *s*__*Parabacteroides_distasonis*, *s*__*Bacteroides_acidifaciens*, and *s*__*Emergencia*_*sp*__*1XD21*_*10.* It is worth noting that the Kruskal–Wallis H test barplot at the species level (Figure 6G) showed that the APS-H group saw a significant decrease in the relative abundance of *Escherichia_coli* in colitis mice and saw an increase in that of *Parabacteroides_distasonis*.

### 3.7. Functional Transformation of APS on Intestinal Microorganisms in DSS-Induced Colitis Mice

**Figure 7:**
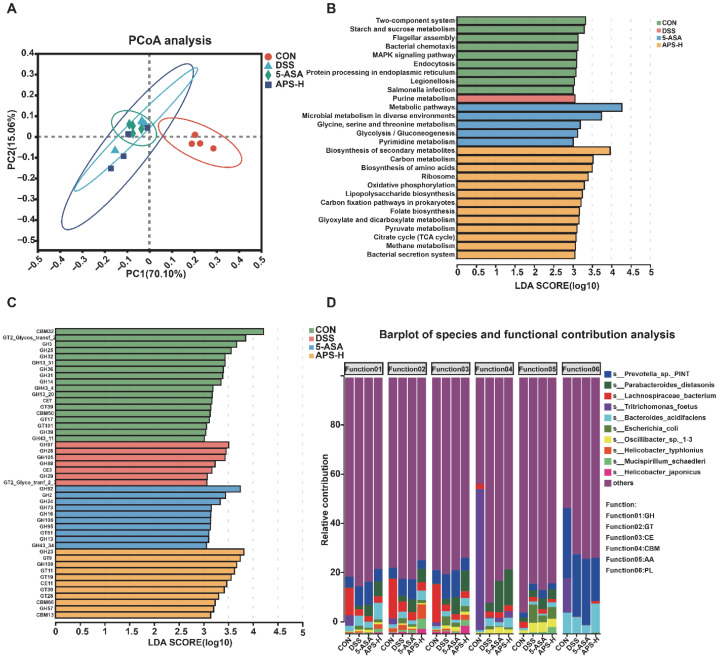
Prophylactic APS administration regulated the functionality of gut microbiota. (**A**) PCoA plot built upon the Kyoto Encyclopedia of Genes and Genomes (KEGG) pathways of recognized bacterial genes. LEfSe examination of variational enrichment of microbial KEGG pathways (**B**) and carbohydrate-active enzyme (CAZymes) (**C**) (LDA > 3). (**D**) The top 10 differentially enriched CAZymes and their functional contributions.

In order to further understand the effects of DSS and APS on the function of the mouse colon microflora, metagenomic sequencing technology was employed. The PCOA map based on KEGG enzymes showed different functional maps among the four groups (similarity analysis [ANOSIM] (*p* < 0.05, Figure 7A). LDA and LEfSe analyses revealed the difference and enrichment of various metabolic pathways in different treatment groups (Figure 7B). It could be seen from the Figure that 13 metabolic pathways were enriched in the APS-H group, among which the LDA values of the biosynthesis of secondary metabolites, carbon metabolism, and the biosynthesis of amino acids are in the top three, indicating that their influence in the APS group was more significant (Figure 7B). We further discovered that several carbohydrate-active enzymes, namely GH23, GT9, GH109, GT11, GT19, CE11, GT30, GT28, CBM66, GH57, and CBM13, were enriched in the APS-H group (Figure 7C). As a matter of fact, *Parabacteroides distasonis* (the top three) are the main contributors in the regulation of GH, GT, CE, and CBM (Figure 7D).

### 3.8. Correlation Analysis of Microflora with Inflammatory Cytokines and Tight Junction Protein

**Figure 8:**
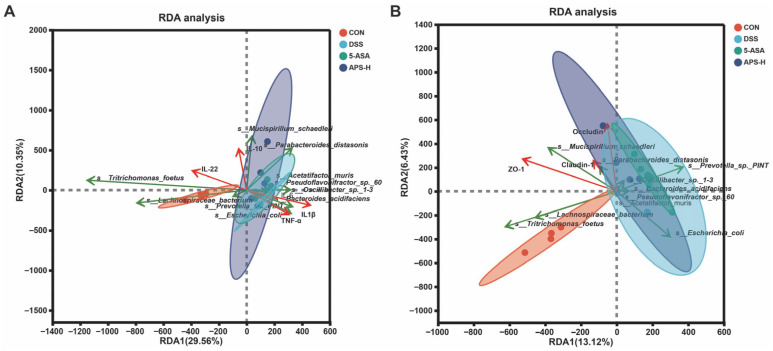
Analysis of redundancy/canonical correlation (RDA/CCA). Analysis of RDA for species-level flora and parameters associated with (**A**) colonic inflammation and (**B**) colonic tight junctions, respectively. Inflammatory cytokines and tight junctions are indicated by red arrows; microbial flora are indicated by green arrows. The length of the arrow connection indicates the degree of correlation between the two. The longer the connection, the greater the correlation; conversely, the shorter the connection, the smaller the correlation. The angles between environmental factors indicate their correlation: acute angles denote positive correlation, obtuse angles indicate negative correlation, and right angles represent no correlation.

The association between microbial flora, inflammatory cytokines, and tight junction proteins was investigated using RDA/canonical correlation analysis (RDA/CCA). The relative abundance of *Lachnospiraceae_bacterium*, *Tritrichomonas_foetus*, *Parabacteroides distasonis,* and *Mucispirillum_schaedleri* had a positive associative relationship with serum anti-inflammatory cytokine level, colon anti-inflammatory cytokine mRNA expression level (Figure 8A), and tight junction function (Figure 8B). *Escherichia_coli*, *Oscillibacter_sp._1*-*3*, *Bacteroides_acidifaciens,* and *Pseudoflavonifractor_sp._60* were negatively correlated with the above indexes.

## 4. Discussion

The primary symptoms of UC are abdominal pain, weight loss, diarrhea, indigestion, and rectal bleeding [20]. In intestinal inflammation research, DSS is commonly used to induce colitis for the establishment of UC models [11]. This study used a 4% DSS-induced mouse UC model, and the mice showed the same symptoms as described above. The colons also displayed an ulcerated surface and a reduced length within identical timing. These typical UC manifestations (both systemic symptoms and colonic pathological changes) confirm that the DSS-induced UC model in mice was successfully established.

Similar to other plant-derived polysaccharides [21,22], our findings showed that APS could significantly alleviate colitis symptoms by lowering body weight loss, improving DAI scores, and lengthening the colon. It is worth noting that the concentration of DSS commonly used to induce UC models in existing studies is 3% or even lower [12], while we adopted a higher concentration. This more robustly demonstrates the potential of APS as a prebiotic for preventing inflammatory bowel diseases. Additionally, the alleviating effect of APS on DSS-induced colitis was partially dose-dependent; in this study, the mice in the APS-H group exhibited even better outcomes than those in the 5-ASA group. Notably, 5-ASA is a commonly used therapeutic drug for UC, and the dose of 5-ASA applied in our experiment was consistent with its clinically common dosage [19]. Earlier research had also shown that APS had improved weight gain by maintaining gut health [23], which further supports the above view.

Inflammatory bowel disease (IBD) is characterized by the impairment of the gut barrier and the dysregulation of gut homeostasis. In mice with UC, the intestinal structure exhibited phenomena such as ruptured crypts, scant villi, enlarged cell spacing, damaged mucosa, a deficiency of goblet cells, and an excessive infiltration of neutrophils [24]. The histo-morphological observations revealed that APS could restore the crypt structures through reducing neutrophil infiltration and alleviating mucosal damage, thereby significantly improving histopathology scores and the morphology of goblet cells destroyed by DSS, which was similar to the findings of relevant studies on other plant polysaccharides [25,26]. One of the main mechanisms involved in colitis function is apoptosis; excessive IECs apoptosis impairs the gut epithelial barrier, resulting in the development of UC [27]. The present research data showed that the number of apoptotic bodies in DSS mice increased significantly. The apoptosis level was markedly diminished after APS treatment in the mice, which was similar to the findings of relevant studies on other plant polysaccharides [28]. A longer colon, a lower histopathological score, a higher goblet cell count, and a lower apoptotic cell count all supported our hypothesis that APS could strengthen the intestinal mucosal barrier by slowing the progression of gut inflammation and tissue damage.

Tight junction protein expression levels are closely related to intestinal integrity and permeability. Research has found that the abnormal expression of tight junction proteins in the intestine of mice with colitis will exacerbate the occurrence and development of colitis [28]. In line with earlier research, our results showed that DSS intervention significantly reduced the expression levels of ZO-1, Occludin, and Claudin-1, and the structure of tight junctions was damaged. Previous reports have shown that polysaccharides decreased apoptosis and tight-junction breakdown in the colon epithelium of mice in DSS-induced colitis [29]. Similarly, in this study, the protein levels of tight junction proteins in colon tissue, such as ZO-1, occludin, and claudin-1, were down-regulated in the DSS-induced colitis mice. Meanwhile, under transmission electron microscopy, there were losses of adherens junctions, tight junctions, desmosomes, and microvilli, which constitute the brush border in the colonic epithelium. All of these were significantly improved by APS.

The mucus layer, made up of a lot of mucin and antimicrobial peptides, is the intestinal mucosa’s biochemical barrier [30]. The integrity of the intestinal milieu and the regular operation of the gut depend on the presence of two essential mucus proteins, MUC2 and MUC5AC [31]. Given the previous findings on body weight, DAI scores, colon length, and intestinal barrier damage, all of which indicated that APS alleviated DSS-induced colitis in a dose-dependent manner, we selected mice in the APS-H group to detect the expression of MUC2 and MUC5AC. According to our data, DSS treatment significantly reduced the expression of MUC2 and MUC5AC, whereas APS-H alleviated DSS-induced colitis and increased the expression of mucins. Our results are consistent with those of other studies [31], which may indicate that APS-H could reduce DSS-induced UC by promoting mucin formation.

TLR4/MyD88/NF-kB pathway activation is intimately linked to the DSS-induced IBD model. The activation of this pathway promotes the production of pro-inflammatory cyto-kines. Inflammatory bowel disease can be effectively treated or alleviated by down-regulating this pathway using a variety of techniques [32]. It has been reported that APS therapy may control the expression of TLR4/MyD88/NF-κB in tissues and cells [14]. The current research also found that when mice were exposed to DSS attacks, their colon tissue had higher amounts of TLR4, MyD88, and NF-κB proteins. However, the increase in the levels of relevant proteins due to DSS treatment was effectively suppressed by APS-H supplementation. The activation of these pathways controls the generation of downstream pro-inflammatory cytokines. Among these, three important pro-inflammatory cytokines—TNF-α, IL-1β, and IL-6—are of fundamental importance in the induction of UC [33]. According to the research results, it was shown that the addition of APS-H could inhibit the levels of TNF-α, IL-1β, and IL-6 in serum and their mRNA expression levels in colon tissue, as well as enhancing the secretion of anti-inflammatory cytokines such as IL-22 and IL-10 in the serum and colon tissue of mice with colitis caused by DSS. Early studies have found that APS blocks the production of TNF-α and IL-6 through the NF-κB signaling pathway and controls the inflammatory mechanism [14], which is in line with the findings of this study. Therefore, we speculate that APS may decrease the secretion of pro-inflammatory cytokines by preventing the initiation of the TLR4/MyD88/NF-κB pathway, which may be the main approach to ameliorate gut inflammation and injury caused by DSS.

In recent years, it has been confirmed that intestinal flora imbalance and UC are related [34]. The incidence of UC and sustainable development is thought to be caused by disorders of the gut microbiota [34]. An increasing amount of evidence indicates that variations in the variety, constitution, and performance of gut microorganisms raise the risk of UC [35]. When compared to the DSS group in the current study, microbiota diversity and richness in the APS-H group showed an increasing tendency. At the phylum level, Bacteroidetes and Firmicutes in the APS-H group showed a downward trend when set against the DSS group. According to earlier research, patients with IBD have an imbalance of the gut microbiota. There is a decreased abundance of *Eubacterium*, *Lactobacillus*, *Ruminococcus*, *Bifidobacterium*, *lachnospiraceae*, and *Balutia species* [36,37], while there is an increased abundance of pathogenic microbes like *Helicobacter*, *Bacteroides*, *Clostridium*, and *Oscillibacter* [38]. Similarly, we discovered that UC mice exhibited an imbalance in gut microbes, with a reduced abundance of *unclassified_f*_*lachnospiraceae*, *Parabacteroides*, *Alistipes*, and *Mucispirillum* and an enrichment of *Bacteroides*, *Prevotella*, *Clostridium*, *Oscillibacter*, *Pseudoflavonifractor*, and *Escherichia* in the DSS group. Furthermore, at the species level, most of the dominant species in DSS tissue samples belong to *Proteobacteria*, while the prevailing species in the 5-ASA and APS-H groups belong to the genus *Parabacteroides*. Additionally, LDA and LEfSe were utilized to analyze the biomarkers in different treatment groups. The distinguishing features in the CON group were the high abundance of previously reported commensals [39], including *s__Lachnospiraceae_bacterium*, *s__Roseburia_sp__CAG_10041_57*, *s__Eubacterium_plexicaudatum*, *s__Roseburia*_sp__*1XD42*_*69,* and *s_Acetatifactor_muris*. The pathogenic species with high LDA scores in the DSS group were *s__Bacteroides_intestinalis*, *s__Bacteroides_thetaiotaomicron*, *s__Faecalibaculum_rodentium*, and *s__Streptococcus_alactolyticus*. APS-H supplementation significantly preserved the abundance of potential probiotic strains [40], including *s__Parabacteroides_distasonis*. Our research findings align with many other investigations. These studies have clearly shown that polysaccharides sourced from various plants possess the ability to alleviate colitis within an animal model induced by DSS [41,42]. It is speculated that this beneficial effect could be linked to the modulation of the gut microbiota.

Although the exact origin of IBD is currently unresolved, the general consensus is that a modified functional mechanism and immune system dysregulation brought on by dysbiosis are most likely to blame [43]. Understanding the connection between functional signatures and microbiome taxonomic levels was our aim. The APS-H group had 13 metabolic pathways that were enriched, according to metagenomic analysis. Of these, the LDA values for carbon metabolism, the biosynthesis of amino acids, and the biosynthesis of secondary metabolites are in the top three, suggesting that their impact on the APS group is greater. Notably, a comparison of enriched pathways reveals distinct differences between APS and 5-ASA, which stem from the distinct properties of the two intervention agents and the specificity of bacterial responses. 5-ASA directly targets bacterial stress-signal transduction pathways, regulating bacterial chemotaxis and stress responses to modulate metabolic homeostasis. In contrast, APS, as a natural macromolecular polysaccharide, is first degraded and transformed by gut bacteria before preferentially activating metabolite synthesis and basic metabolism pathways. This difference leads to regulatory signals concentrating in their respective pathway modules with no overlap, verifying that intervention agents from different sources exhibit specificity in regulating the intestinal metabolic network. More importantly, this finding further supports the unique mode of action of APS, which aligns with its potential as a prebiotic. Additionally, we had further identified that multiple CAZymes were enriched in the APS-H group, including GH23, GT9, GH109, GT11, GT19, CE11, GT30, GT28, CBM66, GH57, and CBM13. It is also interesting to note that, in line with earlier research, the functions of CAZyme, including GH, GT, CE, and CBM, were closely linked to the well-known probiotic *Parabacteroides distasonis* in the current study [44,45]. Notably, intestinal homeostasis is maintained in large part by gut microbiota [46]. *Bacteroides*, *Oscillibacter*, and *Porphyromonas* possess pro-inflammatory traits; moreover, an overabundance of them can inflict harm on the gut mucosa [46,47,48]. Conversely, the role of *Parabacteroides distasonis* is to promote anti-inflammatory impact and aid in intestinal mucosal healing [49]. We observed that the immunoregulatory actions of APS-H, such as inhibiting the production of inflammatory cytokines and promoting the differentiation of tight junction proteins, were closely linked to the therapeutic impact of APS-H on UC. The relative abundance of *Mucispirillum_schaedleri* and *Parabacteroides distasonis* was positively correlated with tight junction function and colon anti-inflammatory cytokine mRNA expression. *Escherichia_coli*, *Oscillibacter_sp._1*-*3*, *Bacteroides*_*acidifaciens,* and *Pseudoflavonifractor*_*sp*._*60* were negatively correlated with the colon anti-inflammatory cytokine mRNA expression level and tight junction function. Our observations of changes in the microbiome’s taxonomic diversity and composition were mostly indicative of its functional capacity, which is in line with other research [50]. As a consequence, it becomes possible to make a speculation that the interplay of the immune system and the intestinal epithelial barrier is regulated by APS-H through the intestinal microflora. In addition, according to the correlation and microbial function analysis, APS-H alters the gut microbiota of mice with colitis, which may be associated with the impact of APS-H on the differentiation of tight junction proteins and the expression of inflammatory cytokines.

Despite these promising findings, this study has several key limitations. First, the chemical structure of the APS used in this study remains unknown. Additionally, the study lacks integrated metabolomics and direct mechanistic validation. Therefore, in subsequent studies, we will chemically characterize the structure of APS to establish its structure–activity relationship, verify the roles of the key bacteria participating in the anti-colitis impact of APS, and clarify the molecular mechanisms by which APS displays its efficacy. At the same time, it is necessary to combine metabolomics to elucidate the “microbiota-metabolite-host” cascade regulatory network.

## 5. Conclusions

In conclusion, APS can alleviate the manifestations of colitis in mice through multiple targets. It mainly improves the manifestations of mouse colitis by relieving the reduction in body weight and reducing the injury to the colon tissue. Moreover, it exerts anti-inflammatory effects by up-regulating the levels of tight junction proteins, increasing the number of goblet cells and mucin production, reducing the number of apoptotic cells, and inhibiting the MyD88/TLR4/NF-κB signaling pathway mediated by gut microbiota, thereby decreasing the injury to the intestinal mucosal barrier function brought on by DSS-induced colitis. In addition, APS-H can also avoid the imbalance of the gut microbiota in mice with DSS-induced colitis, restore the relative abundances of important bacteria (*Parabacteroides distasonis*), and regulate the makeup of the gut microbiota. Therefore, APS may be a viable prebiotic to relieve the microbial imbalance and intestinal barrier dysfunction caused by colitis.

## Data Availability

The data supporting the findings of this study are available at online repositories (https://www.ncbi.nlm.nih.gov/sra/PRJNA1188274, accessed on 24 November 2024) or can be obtained by contacting the corresponding author.

## Supplementary Materials

The following supporting information can be downloaded at: https://www.mdpi.com/article/10.3390/nu17183001/s1, Table S1. Primer sequences used for QRT-PCR analysis.

## Abbreviations

The following abbreviations are used in this manuscript:

AB	Alcian blue
APS	Alfalfa polysaccharide
CAZyme	Carbohydrate-active enzyme
DAI	Disease activity index
DSS	Dextran sulfate sodium
GAPDH	Glyceraldehyde-3-phosphate dehydrogenase
H&E	hematoxylin and eosin
IBD	Inflammatory bowel diseases
IOD	Integrated optical density
KEGG	Kyoto Encyclopedia of Genes and Genomes
LDA	Linear discriminant analysis
LEfSe	Linear discriminant analysis effect size
NR database	Non-Redundant Protein Sequence Database
PCoA	Principal Co-ordinates Analysis
RDA	Redundancy analysis
TEM	Transmission electron microscopy
UC	Ulcerative colitis
5-ASA	5-Aminosalicylic Acid
