# Supplementary material for: Alfalfa Polysaccharide Alleviates Colitis by Regulating Intestinal Microbiota and the Intestinal Barrier Against the TLR4/MyD88/NF-κB Pathway
Source: Nutrients. 2025 Sep 19;17(18):3001. doi: 10.3390/nu17183001 (PMC12472594; doi:10.3390/nu17183001)
Supplement: Supplementary file 1 [file nutrients-17-03001-s001.zip › nutrients-3807304-supplementary.pdf]

Supplementary Data

Table S1 Primer sequences used for QRT-PCR analysis

Gene	Forward primer (5′-3′)	Reverse primer (5′-3′)	NCBI reference sequence
GAPDH	GGTCGGTGTGAACGGATTTG	ATGTAGACCATGTAGTTGAGGTCA	NM_001289726.2
IL-1β	ACTCATTGTGGCTGTGGAGA	TTGTTCATCTCGGAGCCTGT	NM_008361.4
IL-6	AGAGGAGACTTCACAGAGGATACC	AATCAGAATTGCCATTGCACAAC	NM_001314054.1
TNF-α	GCGACGTGGAACTGGCAGAAG	GCCACAAGCAGGAATGAGAAGAGG	NM_001278601.1
IL-10	TTCTTTCAAACAAAGGACCAGC	GCAACCCAAGTAACCCTTAAAG	NM_010548.2
TLR-4	TTCAGAACTTCAGTGGCTGGATT	CCATGCCTTGTCTTCAATTGTTT	NM_138554.5
MyD88	TGGCCTTGTTAGACCGTGA	AAGTATTTCTGGCAGTCCTCCTC	NM_010851.3
NF-κB p65	ACACTGGAAGCACGGATGAC	TGTCTGTGAGTTGCCGGTCT	NM_009045.5
